# Predicting Biochemical and Physiological Parameters: Deep Learning from IgG Glycome Composition

**DOI:** 10.3390/ijms25189988

**Published:** 2024-09-16

**Authors:** Ana Vujić, Marija Klasić, Gordan Lauc, Ozren Polašek, Vlatka Zoldoš, Aleksandar Vojta

**Affiliations:** 1 Department of Biology, Faculty of Science, University of Zagreb, 10000 Zagreb, Croatia; ana.vujic@biol.pmf.hr (A.V.);; 2 Genos Glycoscience Research Laboratory, 10000 Zagreb, Croatia; 3 Faculty of Pharmacy and Biochemistry, University of Zagreb, 10000 Zagreb, Croatia; 4 Department of Public Health, University of Split School of Medicine, 21000 Split, Croatia; 5 Croatian Science Foundation, 10000 Zagreb, Croatia

**Keywords:** IgG, *N*-glycosylation, biochemical parameters, physiological parameters, elastic net, deep learning, cardiometabolic events

## Abstract

In immunoglobulin G (IgG), *N*-glycosylation plays a pivotal role in structure and function. It is often altered in different diseases, suggesting that it could be a promising health biomarker. Studies indicate that IgG glycosylation not only associates with various diseases but also has predictive capabilities. Additionally, changes in IgG glycosylation correlate with physiological and biochemical traits known to reflect overall health state. This study aimed to investigate the power of IgG glycans to predict physiological and biochemical parameters. We developed two models using IgG *N*-glycan data as an input: a regression model using elastic net and a machine learning model using deep learning. Data were obtained from the Korčula and Vis cohorts. The Korčula cohort data were used to train both models, while the Vis cohort was used exclusively for validation. Our results demonstrated that IgG glycome composition effectively predicts several biochemical and physiological parameters, especially those related to lipid and glucose metabolism and cardiovascular events. Both models performed similarly on the Korčula cohort; however, the deep learning model showed a higher potential for generalization when validated on the Vis cohort. This study reinforces the idea that IgG glycosylation reflects individuals’ health state and brings us one step closer to implementing glycan-based diagnostics in personalized medicine. Additionally, it shows that the predictive power of IgG glycans can be used for imputing missing covariate data in deep learning frameworks.

## 1. Introduction

Immunoglobulin G (IgG) is one of the best-studied glycoproteins in humans. Both functional domains of IgG, the fragment antigen-binding (Fab) region and the fragment crystallizable (Fc) region, undergo glycosylation which significantly impacts IgG function. Glycosylation of a conserved *N*-glycosylation site Asn-297, present at both heavy chains of the Fc domain, influences the antibody’s conformation, stability, and interaction with Fcγ receptors, thereby acting as a switch between pro- and anti-inflammatory responses [1,2,3,4,5]. The Fab region lacks a conserved glycosylation site; however, 15–30% of IgG molecules contain one or more *N*-glycosylation sites at this region introduced by somatic hypermutation [6,7,8,9]. Fab glycosylation impacts antigen-binding affinity and serum half-life [1,7,8]. Although IgG glycome demonstrates low variability within an individual under homeostatic conditions, disruptions to this equilibrium, such as those occurring during disease or aging, prompt striking changes in the IgG glycome profile [1,6]. However, the IgG glycome exhibits substantial inter-individual variability that is driven by the intricate interplay between an individual’s genetic makeup, epigenetic regulation, and environmental influences [10,11,12].

Studies indicate that changes in the IgG glycosylation profile, particularly a decrease in IgG galactosylation, reflect the underlying inflammatory processes that take place in numerous diseases [6]. IgG glycan changes in diseases like galactosemia and congenital disorders of glycosylation (CDG) are directly linked to known defects in carbohydrate metabolism, glycan synthesis, or glycan modification pathways [13,14]. However, for most diseases, especially multifactorial ones, the relationship between changes in IgG glycosylation and disease is complex and influenced by numerous factors. In complex diseases, genetics, environmental influences, and metabolic influences interact at multiple levels, and due to their plasticity, changes in IgG glycans reflect these influences [6]. It remains unclear whether changes in the IgG glycome are involved in disease onset or occur as a response to pathological processes within the organism. Nevertheless, the fact that the IgG glycome is affected by various physiological and pathological states makes it a promising biomarker for a person’s overall health status [1]. Observation that IgG glycans can estimate biological age led to the development of the glycan clock, a biological age biomarker based solely on IgG glycosylation [15]. Integration of clinical traits further enhanced age prediction models [15,16]. Numerous studies have now confirmed the association between the IgG glycome and various clinical traits associated with disease states [17,18,19,20,21,22,23,24,25,26,27]. These studies have shown correlations between specific IgG glycan traits and lipid metabolism, with specific glycans linked to levels of high-density lipoprotein (HDL) cholesterol, low-density lipoprotein (LDL) cholesterol, total cholesterol, and triglycerides [21,24]. Beyond lipids, accumulating evidence suggests a role for IgG glycosylation in blood pressure regulation. Studies have identified associations between altered IgG glycosylation and prehypertension, established hypertension, and both systolic and diastolic blood pressure (SBP and DBP). Additionally, specific glycan features have been shown to predict incident hypertension [18,20,25,27]. The repertoire of biochemical and physiological traits associated with IgG glycome is continually expanding through ongoing research. In addition to previously established associations, recent studies have revealed connections with serum levels of glucose, insulin [21], hemoglobin A1c (HbA1c), fibrinogen, D-dimer [15], uric acid [17], and creatinine [28]. However, a question remains: do changes in IgG glycosylation represent a cause or consequence of these metabolic imbalances? To address this, Meng et al. employed a bidirectional Mendelian Randomization (MR) analysis. By integrating GWAS data for metabolic traits and IgG glycans, along with IgG *N*-glycan-QTLs and metabolic-QTLs, their study confirmed the existence of bidirectional causality between IgG glycosylation and various metabolic traits [26].

The IgG glycome exhibits substantial biomarker potential as evidenced by numerous studies leveraging its attributes to construct predictive models for various diseases [1]. Given that studies demonstrated both association and bidirectional causality between IgG glycans and metabolic traits, we hypothesize that IgG glycan data holds potential for predicting different biochemical but also physiological parameters. This study used data from the Korčula cohort to build predictive models for biochemical and physiological parameters using IgG glycans as the input feature and data from an additional cohort (the Vis cohort) for validation of the results. We employed two distinct methods, elastic net and deep learning, to evaluate the predictive power of IgG glycans. Establishing this link would support the notion that the IgG glycome profile reflects an individual’s metabolic health status. Furthermore, the ability to predict biochemical and physiological parameters from IgG glycans holds additional value, as it could facilitate imputation of missing biochemical and physiological data in datasets intended for further analysis. In this study, we assessed the predictive capacity of IgG glycans for imputing missing covariate values, including selected physiological and biochemical parameters with a focus on cardiometabolic health.

## 2. Results

### 2.1. Population Characteristics

Baseline information of all study subjects in both the Korčula and Vis cohorts, including information regarding biochemical and physiological traits used in this study, is summarized in Table 1.

### 2.2. Glycomics Data Correlation

**Figure 1:**
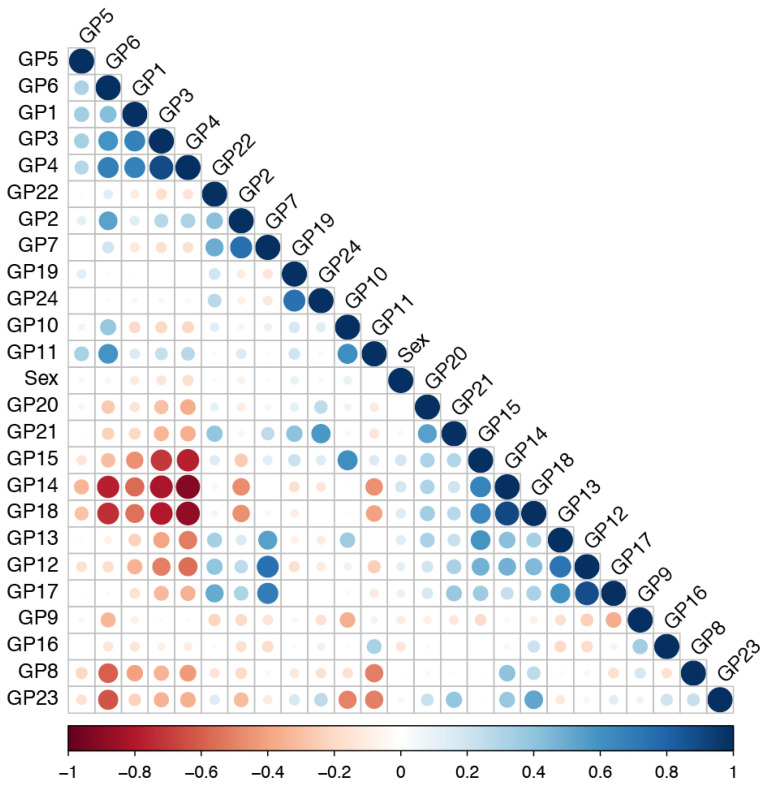
Correlation between the glycan peaks in the glycomics data. The dataset shows high correlation between glycans, which necessitates appropriate predictive models that are immune or at least resistant to such correlated data. Both the elastic net and deep learning have that required property. Glycan composition of each peak within the human IgG glycome has been previously established [32]. A representative chromatogram of the human IgG glycome and a detailed characterization of the glycan composition for each peak can be found in Pučić et al. [32] and Krištić et al. [15].

The Glycomics data show strong correlations within several clusters of glycan peaks (GP) and a weak correlation with biological sex (Figure 1). Positively correlated glycan peaks are mostly located on the same part of the IgG chromatogram, such as peaks GP3 and GP4 or GP14 and GP15. Earlier eluting peaks harbor less complex glycan structures that mostly lack galactose while later eluting peaks exhibit more complex glycan structures containing one or two galactose residues capped with sialic acid. Later glycan peaks show negative correlations with peaks at the beginning of the chromatogram as seen between peaks GP6 and GP23. This is likely because the increase in abundance of complex glycan structures coincides with a decrease in the number of less complex structures, and vice versa. Correlation analysis of glycomics data demonstrates the need for using regression and machine learning models robust to this type of data and led to the selection of elastic net [29] as a regression model and deep learning [30,31], ultimately linked to the TensorFlow library, as a machine learning model. In contrast, other methods, such as random forests and simple regression, would not be appropriate for generalization and building reliable predictive models on such correlated data.

### 2.3. Prediction of Biochemical and Physiological Parameters from the IgG Glycome Composition

**Figure 2:**
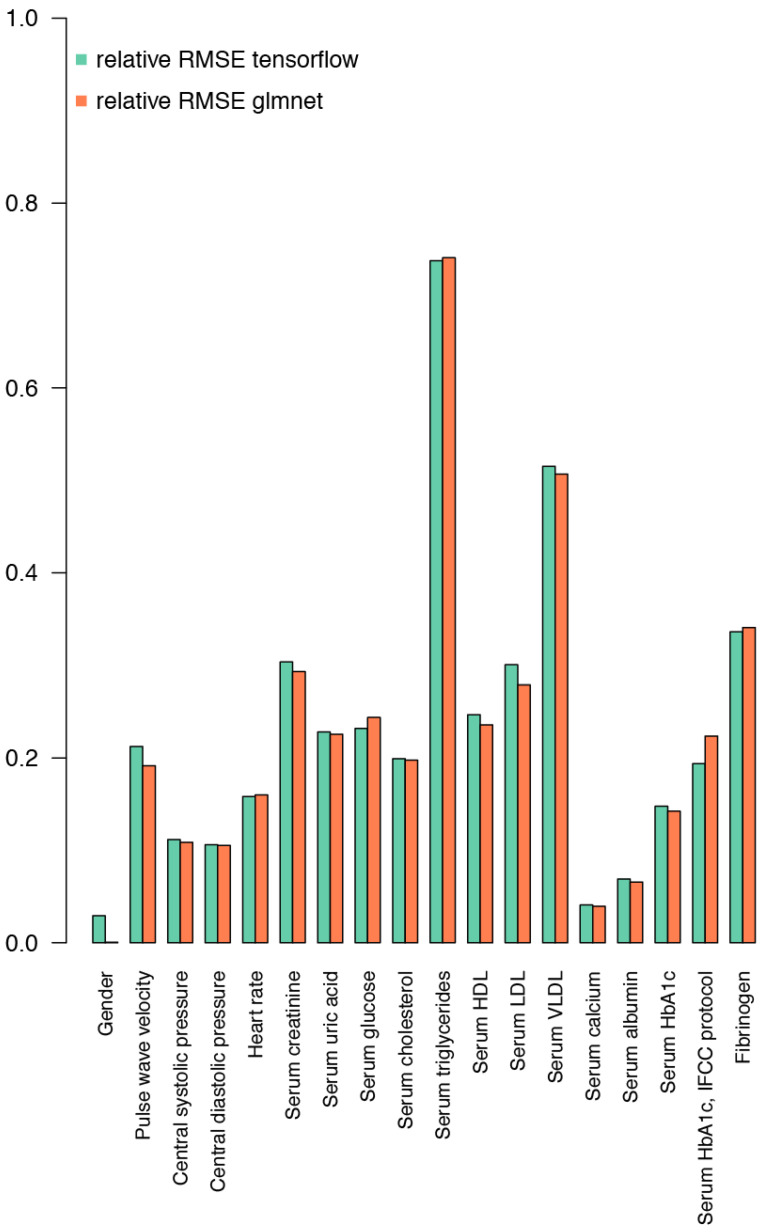
Relative RMSE (root mean square error) normalized to the mean of the dataset for prediction of selected biochemical and physiological parameters based on IgG glycosylation in the Korčula cohort. In addition to the relative area under each of the 24 glycan peaks, only sex was used as an input variable. It is predicted with 100% accuracy, which was used as a “positive control”. Other biochemical and physiological parameters can be predicted from an IgG glycosylation pattern with varying degrees of accuracy. A regression model (glmnet) and a machine learning approach (tensorflow) show a similar degree of predictive power.

IgG glycosylation data, along with sex, have substantial predictive power for certain biochemical and physiological parameters, which can be seen in Figure 2. The performance is variable across the parameters, yet some physiologically relevant values can be inferred from the glycosylation data. These include parameters involved in cardiovascular events: pulse wave velocity, central SBP, central DBP, heart rate, serum creatinine, serum uric acid; parameters involved in lipid metabolism: serum triglycerides, serum cholesterol, serum HDL, serum LDL, serum VLDL; and parameters involved in glucose metabolism encompassing serum glucose and serum HbA1c.

**Figure 3:**
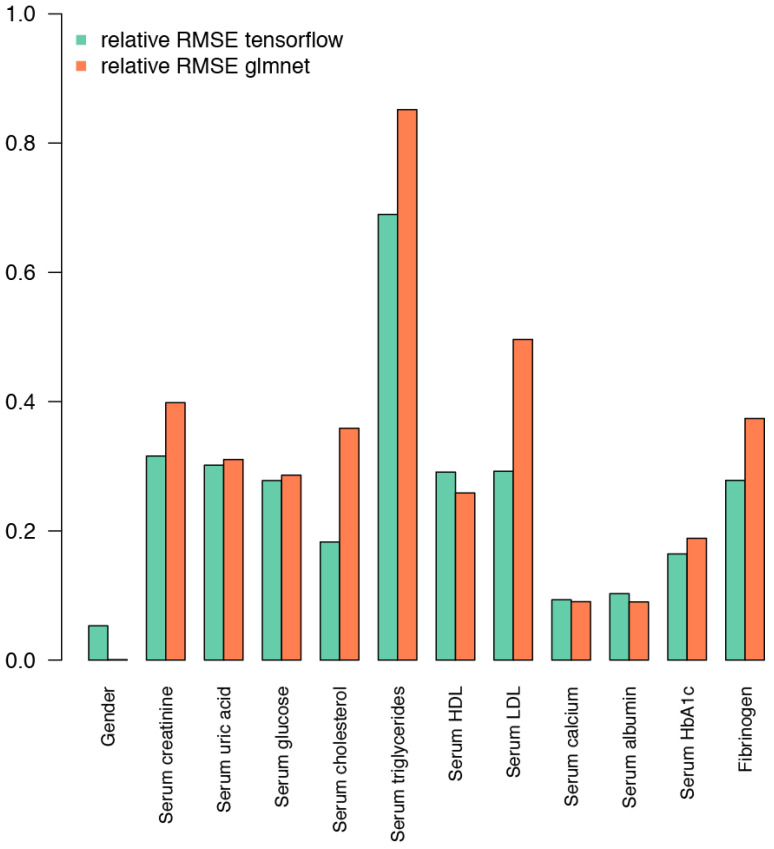
Validation of the model trained on the Korčula cohort in the Vis cohort shows that the model performs reasonably well with the data from an unrelated sampling event that encompasses a different population, time, and batch. Only the parameters measured in both cohorts are reported. Again, relative RMSE normalized to the mean of the dataset is shown as a metric of the success of prediction for selected biochemical and physiological parameters (lower RMSE means better prediction), based on IgG glycosylation as input. The machine learning approach (tensorflow) performs significantly better for most parameters than the regression model (glmnet). The machine learning approach has the additional advantage of being easily extensible.

Performance on both the Korčula and the Vis cohorts was used to select hyperparameters for deep learning. Results on the Korčula cohort with 16% observations set aside for testing and using the Vis cohort for testing were in agreement for all the combinations of hyperparameters tested. Adding or removing hidden layers, changing the number of units per layer, changing the activation function, and changing the number of epochs gave inferior results compared to the selected hyperparameters. This architecture of the deep learning network is therefore close to the optimum for the information contained in the glycans. To compare the performance of deep learning and elastic net models, we normalized the RMSE of predicted biochemical and physiological parameters by the mean of each respective parameter and plotted the relative RMSE values on graph (Figure 2 and Figure 3). Although the performance of the deep learning model is similar to the elastic net regression when validated on the data from the same cohort (Figure 2), it shows superior performance when compared to elastic net regression in the case of validation on the Vis cohort (Figure 3).

While validation on a subset of the same cohort, Korčula cohort, containing a larger set of physiological and biochemical parameters, gave valuable insight into the performance of the model and enabled optimization, a better insight into the power of a model to generalize was given when it was applied on the Vis cohort, where the same sample collection and glycan analysis protocols enabled direct comparison, yet the dataset was collected from a different population and at a different time (Figure 2 and Figure 3).

When we observe each parameter separately (Figure 2 and Figure 3) we can see that IgG glycan composition offered the strongest predictive power for serum albumin levels, followed by serum uric acid levels, in both cohorts. In the Korčula cohort, our glycan-based models also demonstrated superior performance in predicting central DBP and SBP. Conversely, IgG glycan composition exhibited the weakest predictive ability for serum triglycerides in both cohorts and serum VLDL levels in the Korčula cohort only.

Prediction of an individual’s sex, which was used as an input variable, was an additional “sanity check” which any considered model needed to pass. All models we discussed correctly classified all samples by sex.

## 3. Discussion

In this study, we demonstrated the potential of IgG glycosylation data in predicting biochemical and physiological parameters. While the models exhibited some variability across different parameters, specific biochemical and physiological traits were successfully inferred. These included those related to lipid and glucose metabolism, as well as cardiovascular disease risk. This finding aligns with previous research demonstrating associations between IgG glycosylation patterns and various metabolic disorders, traits, and cardiovascular events [21,24,25,33,34]. Our IgG glycan-based models achieved strong predictive performance for serum albumin levels in both cohorts. This is particularly interesting because low serum albumin (hypoalbuminemia) is recognized as a risk factor for all-cause, cancer, cardiovascular, and respiratory mortalities [35,36,37,38]. Additionally, decreasing albumin levels have been associated with increasing age [39]. Intriguingly, IgG glycosylation, particularly Fc monogalactosylation, has also been linked to life expectancies in both males and females [40]. Furthermore, several IgG glycans have been reported to change with chronological and biological age, with most extensive changes including galactosylation [15]. The fact that our IgG-glycan-based models are able to predict serum albumin levels, a well-known mortality risk predictor, suggests that IgG glycosylation itself may hold promise as a potential prognostic factor in an individual’s mortality risk.

Plomp et al. [21] demonstrated that for all IgG subclasses, reduced galactosylation alongside increased core fucosylation are associated with increased inflammation, assessed by the levels of inflammatory marker CRP, low serum HDL cholesterol, and high triglycerides, which are all indicators of poor metabolic health [21]. Further analysis of IgG glycan structures revealed significant correlations between specific glycan peaks and total cholesterol, triglycerides, and LDL cholesterol levels, with nine glycan peaks, including GP1, GP4, GP5, GP6, GP11, GP14, GP18, GP20, and GP21, used to build a model capable of differentiating individuals with dyslipidemia from healthy controls [24]. In our study, serum levels of HDL, LDL, and total cholesterol were successfully inferred from IgG glycosylation data in both cohorts, further supporting the potential link between IgG glycosylation and lipid metabolism.

In addition to lipids, our models exhibited the ability to predict serum glucose and HbA1c levels in both the Vis and Korčula cohorts. Alterations in IgG glycosylation have been linked to impaired glucose metabolism in both type 1 (T1DM) [41] and type 2 (T2DM) diabetes mellitus [34]. Subclass-specific IgG Fc glycan changes, including increased monogalactosylation, agalactosylation, and decreased sialylation and bisecting GlcNAc in IgG4; increased bisecting GlcNAc and decreased digalactosylation and total galactosylation in IgG2; and increased monogalactosylation in IgG1, were associated with T2DM [34]. Another subclass-specific study revealed negative associations between insulin levels and IgG1 fucosylation, IgG4 galactosylation, and sialylation, while IgG4 bisection correlated positively with glucose levels. Both insulin and glucose are recognized markers of T2DM [21]. In pregnant women, metabolic parameters related to insulin levels and insulin resistance were significantly associated with core fucosylated, bisected (FA2B), and afucosylated, disialylated (A2G2S2), glycan structures [19].

Hypertension, along with T2DM, is a risk factor for cardiovascular disease. Several studies showed that incorporating IgG glycan data into prediction models improves risk assessment for hypertension compared to traditional models based on age, gender, and BMI [18,25,27]. For instance, in the Kazakh population, decreased galactosylation and increased fucosylated agalactobiantennary Fc glycans in all IgG subclasses correlated with higher SBP and DBP. A model incorporating nine specific IgG glycan traits successfully discriminated between hypertensive and healthy individuals [25]. Additionally, study on Uygur, Kazak, Kirgiz, and Tajik populations revealed ten subclass-specific IgG Fc glycan traits, primarily reflecting decreased galactosylation and sialylation, significantly associated with hypertension. A glycan-based model with five selected glycan traits showed moderate improvement in prediction [18]. Moreover, Kifer et al. identified four specific IgG glycan traits, including bisecting GlcNAc, GP4, GP9, and GP21, predictive of incident hypertension. A linear combination of these traits correlated with both incident hypertension and both SBP and DBP [27]. Central SBP and DBP are among the parameters for which IgG glycan composition showed the best predictive potential in our study. Emerging evidence suggests central blood pressure is more predictive of future cardiovascular events than brachial pressure [42].

Hyperuricemia, a metabolic condition marked by elevated serum uric acid levels, contributes to occurrence of both hypertension [43] and T2DM [44]. Interestingly, one study identified six specific IgG glycan traits positively and another six negatively correlating with serum uric acid levels. Furthermore, including two specific IgG glycan traits in a model alongside BMI and gender distinguished individuals with hyperuricemia from healthy controls [17]. Notably, our IgG glycan-based models achieved strong predictive performance for serum uric acid levels in both the Korčula and Vis cohorts. Barrios et al. [45] proposed that IgG glycans also have a role in kidney function. They identified 14 IgG glycan traits associated with renal function, encompassing galactosylation, sialylation, and bisecting *N*-acetylglucosamine levels [45]. Moreover, a study using a lectin microarray for IgG glycosylation profiling in peripheral artery disease found a positive association between creatinine levels and IgG fucosylation and galactosylation [28]. Elevated serum creatinine indicates impaired kidney function. In our study, models incorporating IgG glycan data successfully predicted serum creatinine levels in both cohorts, further supporting the potential role of IgG glycosylation in kidney function.

Both IgG glycan-based models developed in this study demonstrated their predictive potential for various biochemical and physiological traits known as risk factors for cardiometabolic diseases. Our study further emphasizes the utility of IgG glycosylation as a valuable marker of an individual’s overall health status. This work also shows that analyzing IgG glycosylation in cohorts with other response variables like diseases or medical conditions may be useful as a step toward glycan-guided diagnostics, using protein glycosylation, a powerful but often overlooked tool, as a substantial upgrade to today’s state of the art in personalized medicine. In those more complex scenarios, we envision that deep learning may significantly outperform classical regression methods due to its ability to capture the complexity of the glycosylation process and the intricate interactions in health and disease. Current study clearly demonstrates that salient information about an individual’s health status is encoded in the protein glycosylation pattern, though extracting it and utilizing its full potential requires large cohorts and sophisticated data analysis methods.

Highly correlated data, such as glycans, have to be analyzed carefully using models that are relatively robust to correlated data. We decided to use elastic net regression, a hybrid between the LASSO and Ridge regression, as a benchmark representing established methodology with robustness to correlated data [29]. In an attempt to build a model that performs at least at the same level and possesses the robustness to correlated data yet is extensible and easily adjusted to other response variables [46], such as disease state, we turned to deep learning using the “keras” library, which taps into the TensorFlow library [47]. The deep learning model demonstrated its superiority when presented with a different cohort for validation (Figure 3). This clearly demonstrates that a deep learning network generalizes better than an elastic net model. A deep learning model, having demonstrated its usefulness in extracting information from the IgG glycosylation profile data, can be easily modified to detect disease status when presented with a dataset containing the appropriate response variable, i.e., the disease state or stage. This is important when looking beyond inferring simple biochemical or physiological parameters. The key useful property of the deep learning model is its ability to capture the complexity of the glycomics data and build accurate predictive models if the response variable shares significant information with the IgG glycosylation pattern, which is often the case with a large number of diseases and conditions [1].

Missing data is a prevalent issue across diverse research fields, such as clinical studies, microarray and spectrometric datasets, and OMICS studies, including glycomics, epigenomics, and genomics [48,49,50,51]. If not handled properly, missing data can significantly compromise studies, reducing statistical power, introducing bias in parameter estimates, and undermining sample representativeness. When managing missing data, imputation methods are favored over complete case analysis or listwise deletion [52]. Deep learning-based models have emerged as powerful tools for missing value imputation. Due to their enhanced imputation accuracy and ability to handle intricate missing patterns and data structures, deep learning-based models have demonstrated superiority over other established imputation methods [53]. This study suggests that the predictive capacity of IgG glycans could be leveraged by researchers to integrate IgG glycome data into deep learning frameworks for imputing missing covariate values, including biochemical and physiological traits themselves. The model can easily be extended to extract the data from IgG glycosylation profiles and leverage that data for detecting the disease status in subsequent research.

## 4. Materials and Methods

### 4.1. Cohorts

This study was performed using previously published data obtained from two cohorts—the Korčula cohort (950 individuals) [54,55] and the Vis cohort (888 individuals) [55,56,57]. The Korčula cohort belongs to an adult population of the island of Korčula, Croatia, where all subjects were aged 18 and over [54,55]. All respondents gave informed consent before participating and the study followed all relevant ethical regulations. The Vis cohort belongs to an adult population (also 18 and over) of the Croatian island Vis who were recruited within a larger genetic epidemiology program with the goal to investigate genetic variability and map genes influencing common complex diseases and disease traits in genetically isolated populations [55,56,57]. As for the Korčula cohort, this study also followed all relevant ethical regulations and all respondents signed an informed consent form before participating. Studies in which data from the Korčula and Vis cohort were published received ethical approval from the University of Split, School of Medicine, and the South East Scotland Research Ethics Committee.

### 4.2. Biochemical and Physiological Parameters

For this study we used previously collected data for physiological and biochemical parameters from individuals in the Korčula [54,55] and Vis cohorts [55,56,57]. The parameters measured in both cohorts included serum creatinine, serum uric acid, serum glucose, serum cholesterol, serum triglycerides, serum HDL, serum LDL, serum calcium, serum albumin, serum HbA1c, and fibrinogen. Additional parameters were assessed only in the Korčula cohort due to their unavailability in the Vis cohort. These included pulse wave velocity, central SBP, central DBP, heart rate, and serum VLDL.

### 4.3. IgG Isolation and N-Glycan Analyses

For the prediction of physiological and biochemical parameters, we used existing IgG glycosylation data from the Korčula and Vis cohorts published in Trbojević-Akmačić et al. [58] for the Korčula cohort and Pučić et al. [32] for the Vis cohort. Briefly, IgG was isolated from human plasma by CIM^®^ r-Protein G LLD 0.2 mL Monolithic 96-well Plate (2 µm channels) (BIA Separations, a Sartorius company, Ajdovščina, Slovenia). Plasma samples were diluted with 1xPBS (pH 7.4) in a 1:7 ratio for the Korčula cohort and 1:9 for the Vis cohort. Diluted samples were applied to preconditioned protein G plates. To remove unbound proteins, plates were washed with 1xPBS (pH = 7.4). Bound IgG was eluted from the protein G plates with 0.1 M formic acid (pH = 2.5) and immediately neutralized to pH 7.0 using 1 M ammonium bicarbonate. Dried IgG eluates were denatured with 1.33% sodium dodecyl sulfate (SDS) and glycans were released by digestion with recombinant *N*-glycosidase F (PNGase F). Released glycans were labeled with 2-aminobenzamide (2-AB). Fluorescently labeled IgG glycans were analyzed using ultra-high-performance liquid chromatography based on hydrophilic interactions and fluorescence detection (HILIC-UHPLC-FLD). Data was processed using an automatic processing method with a traditional integration algorithm. The IgG glycans’ chromatograms were integrated into 24 peaks where the amount of glycans in each peak was expressed as a percentage of the total integrated area (% area). Glycan structures corresponding to each peak were determined by exoglycosidase digestion and mass spectrometry as described in Pučić et al. [32].

### 4.4. Building of Predictive Models Using IgG Glycan Data

A cohort of 950 individuals with complete sets of observations for IgG glycans was used to predict selected biochemical and physiological parameters (the Korčula cohort). An additional cohort of 888 complete observations (the Vis cohort) was used for validation of a subset of biochemical and physiological parameters present in both cohorts. The area (expressed numerically as a percentage, range 0 to 100) under each of the 24 glycan peaks (composition determined by mass spectrometry) and the sex of the individual were used as inputs. Glycomics data were transformed by taking the natural logarithm.

Analysis was performed in an R statistical environment [59]. Package “corrplot” [60] was used for visualization of correlations in the glycomics data. Elastic net regression was performed using the “glmnet” package [61]. For deep learning, we used the “keras” library [62]. The deep learning network consisted of an input layer, three hidden layers of 24 units and an output layer (single unit), with the “relu” activation function; training was performed over 24 epochs with 1/4 of the data set aside for the validation set. Before training, 16% of all observations were set aside as the testing set, on which all performance measurements were conducted, except when the Vis cohort was used for testing/validation of the biochemical and physiological parameters present in both cohorts (the complete Korčula cohort was used for training in that case). Hyperparameters for deep learning were selected manually [63] by systematically varying the number of hidden layers (two to six), number of units in each layer (8, 16, 24, 32, 64), activation function (“relu”, “selu” and combinations thereof), and the number of epochs (10, 24, 50, 100, 500).

## Figures and Tables

**Table 1:** Demographic characteristics, biochemical and physiological parameters measured in the Korčula and Vis cohorts. The table presents the number of participants categorized by sex, along with the mean and standard deviation (SD) for each parameter. Missing data points for the Vis cohort are denoted by “/”. Nonparametric statistics are used to compare data from two cohorts. HDL (High-density lipoprotein), LDL (Low-density lipoprotein), VLDL (Very-low-density lipoprotein), and HbA1c (Hemoglobin A1c).

	Korčula Cohort (N = 950)	Vis Cohort (N = 888)	*p* Value
Female	615 (64.7%)	521 (58.7%)	
Male	335 (35.3%)	367 (41.3%)	
Age (years)	53.76 ± 16.97	56.30 ± 15.74	
Pulse wave velocity	8.64 ± 2.24	/	
Central systolic pressure	120.16 ± 16.72	/	
Central diastolic pressure	81.51 ± 9.20	/	
Heart rate	65.64 ± 10.34	/	
Serum creatinine	82.70 ± 17.37	87.73 ± 28.37	2.064 × 10^−6^
Serum uric acid	289.28 ± 79.20	310.83 ± 93.19	5.564 × 10^−7^
Serum glucose	5.68 ± 1.48	5.73 ± 1.55	4.201 × 10^−1^
Serum cholesterol	5.81 ± 1.22	6.06 ± 1.14	4.405 × 10^−6^
Serum triglycerides	1.39 ± 0.86	1.59 ± 1.01	7.655 × 10^−7^
Serum HDL	1.49 ± 0.36	1.60 ± 0.35	1.031 × 10^−10^
Serum LDL	3.70 ± 1.05	3.81 ± 1.05	2.736 × 10^−2^
Serum VLDL	0.63 ± 0.36	/	
Serum calcium	2.43 ± 0.10	2.33 ± 0.16	2.051 × 10^−83^
Serum albumin	45.04 ± 2.91	45.01 ± 3.64	2.660 × 10^−1^
Serum HbA1c	5.45 ± 0.77	5.46 ± 0.87	6.659 × 10^−1^
Serum HbA1c, IFCC protocol	36.14 ± 8.42	/	
Fibrinogen	2.79 ± 0.89	3.73 ± 0.99	1.921 × 10^−89^

## Data Availability

This study uses already published data with given studies referenced in the Materials and Methods. The used data is not publicly available but is instead available from the corresponding authors of given studies on reasonable request and in line with the consent given by participants.
